# Supplementary material for: A protocol to automatically calculate homo-oligomeric protein structures through the integration of evolutionary constraints and NMR ambiguous contacts
Source: Comput Struct Biotechnol J. 2019 Dec 26;18:114–24. doi: 10.1016/j.csbj.2019.12.002 (PMC6961069; doi:10.1016/j.csbj.2019.12.002)
Supplement: Supplementary data 1 [file mmc1.docx]

**SUPPLEMENTARY INFORMATION**

The python script to perform the protocol can be downloaded at the following [**LINK**](https://github.com/davidesala/ecnmr)**.**

**Supplementary figures**


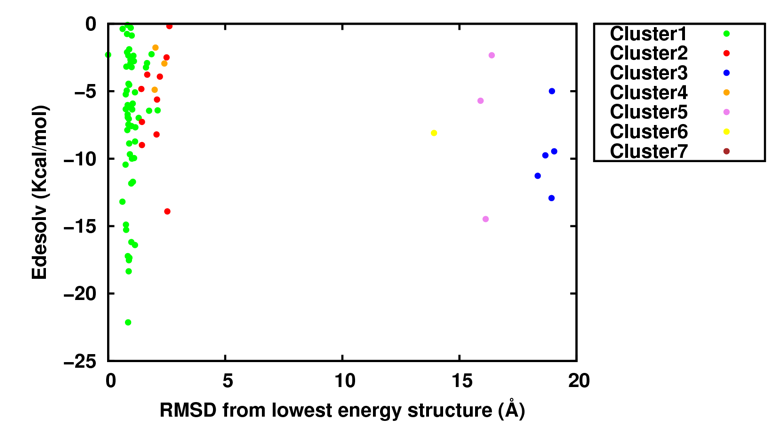


**Figure S1.** Cluster distribution based on the desolvation energy in the first docking run of L-asparaginase II. The colors of the clusters are the same as in Figure 1.


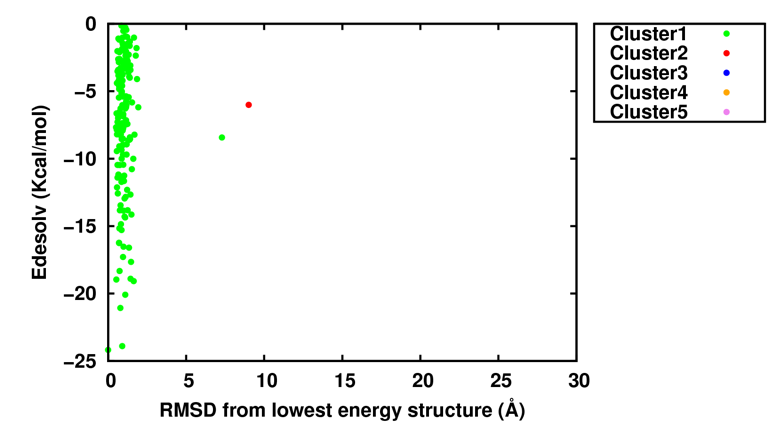


**Figure S2.** Cluster distribution based on the desolvation energy in the second docking run of L-asparaginase II.


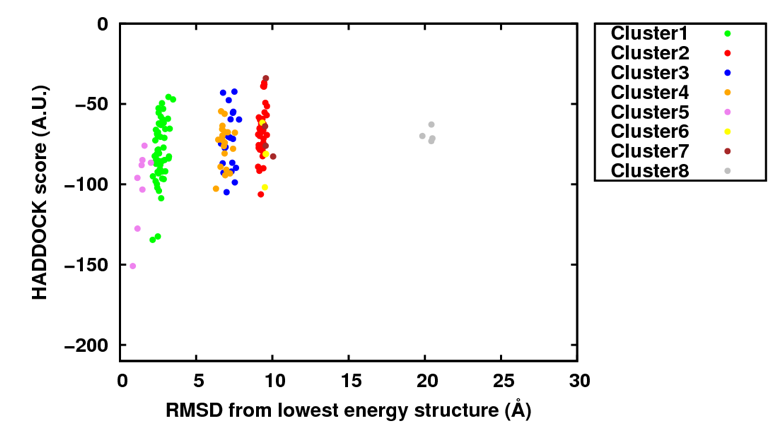


**Figure S3** L-asparaginase II clusters distribution obtained from a monomer-monomer docking run performed using the AIRs violated in the third cluster of the first run

**Figure S4.** Superimposition on the chain A (in green) of the best L-asparaginase II models. **A**) Model 13 AC dimer is in blue and model 6 AD dimer in yellow. **B**) Mode13 AC dimer is in blue and model 15 AD dimer in yellow.


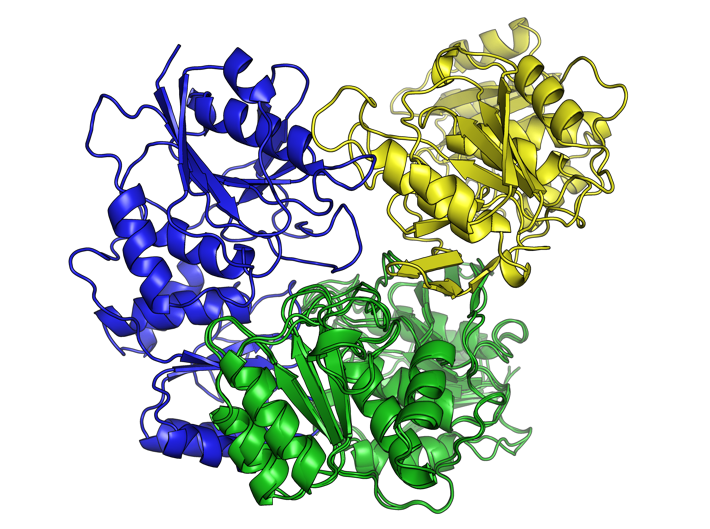

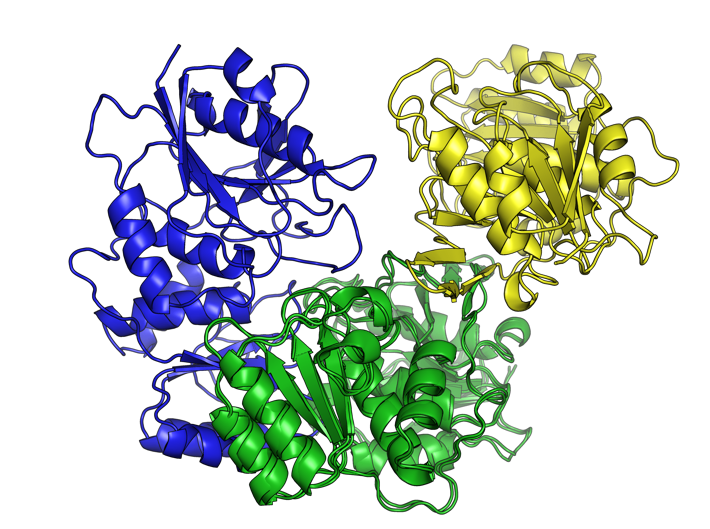


**A**

**B**


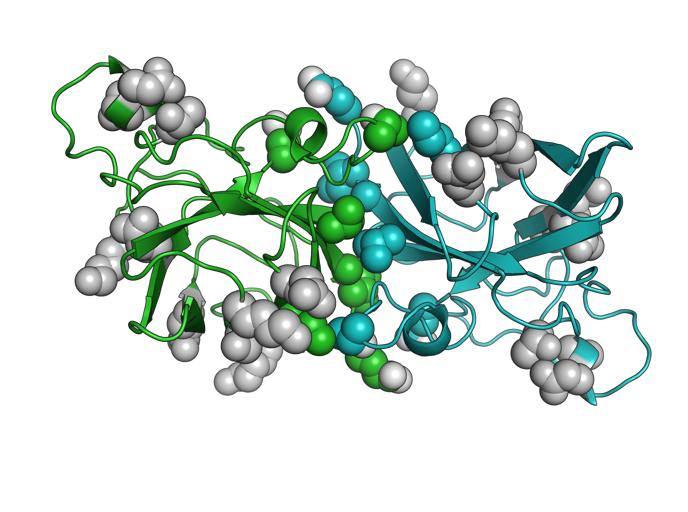


**Figure S5.** Residues used as AIRS in the docking run of Sod1. Residues forming contacts across the interface are colored as the backbone.

**Figure S6.** Fitting of the best model of the clusters 1 and 2 on the Sod1 crystal structure. **A**) cluster 2 in red. **B**) cluster 1 in green

**A**

**B**


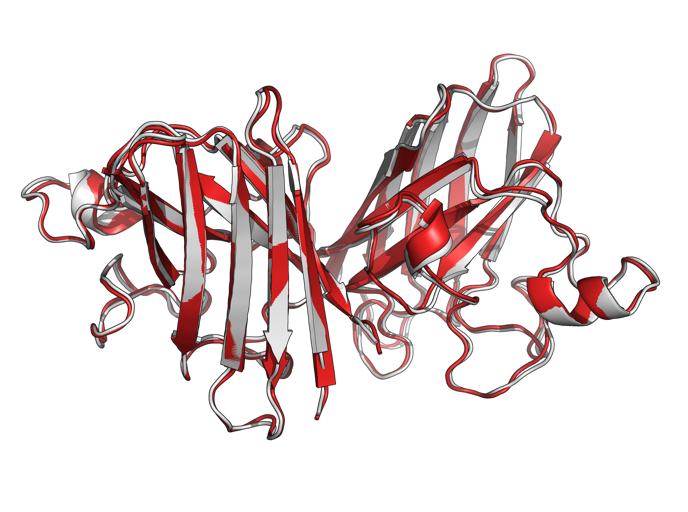

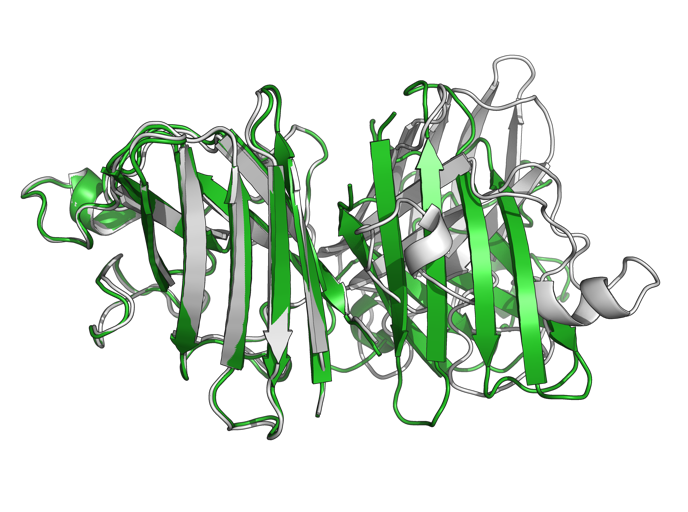

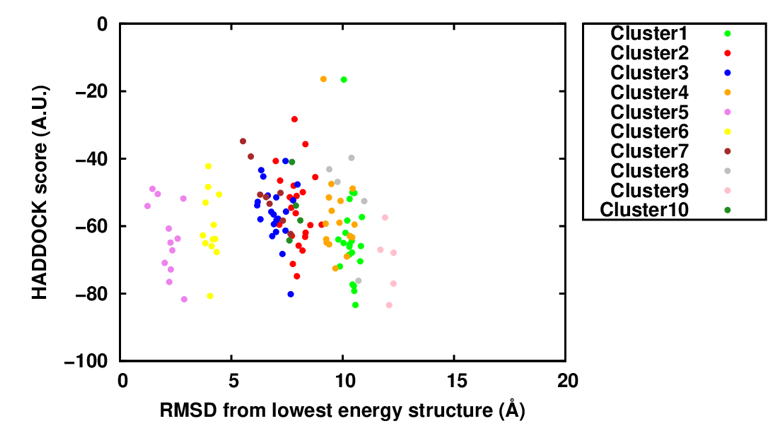

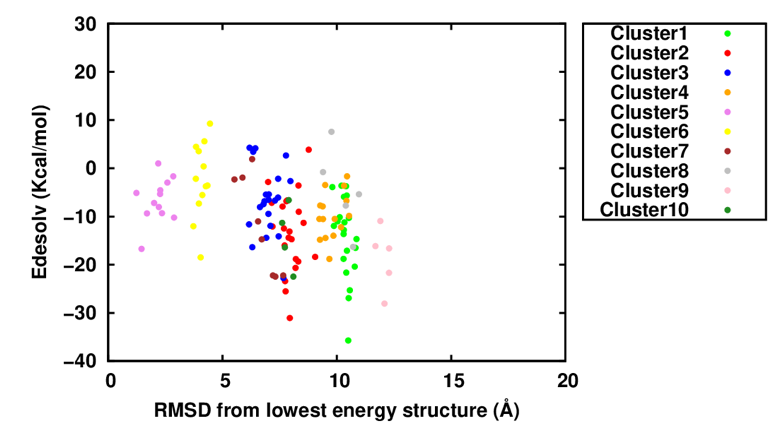


**Figure S8.** Clusters distribution of the Sod1 docking calculation with ECs only at P 0.75. **A**) HADDOCK score. **B**) Edesolv scoring function.


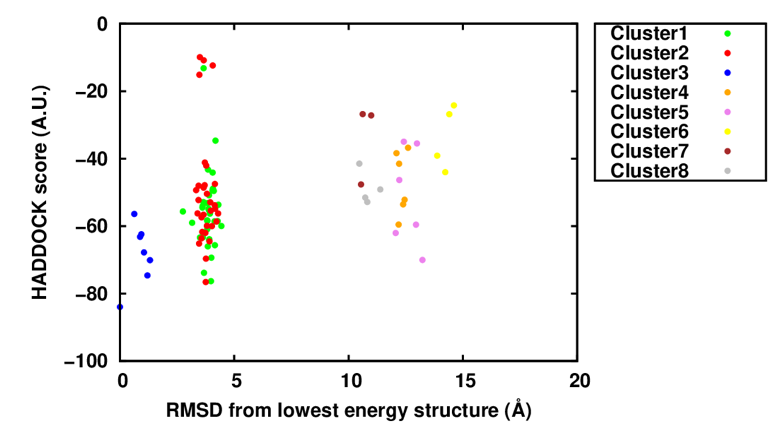

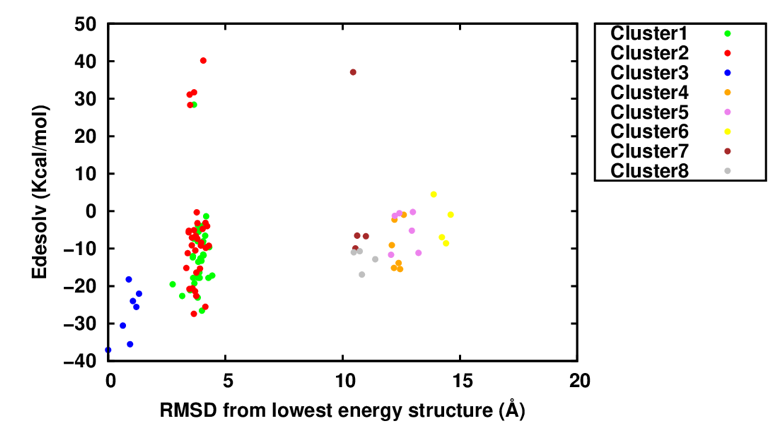


**Figure S9.** Clusters distribution of the Sod1 docking calculation with ECs only at P 0.60. **A**) HADDOCK score. **B**) Edesolv scoring function.


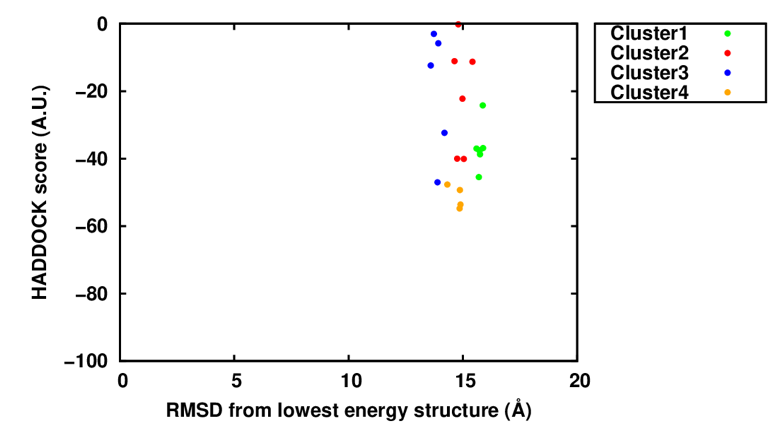

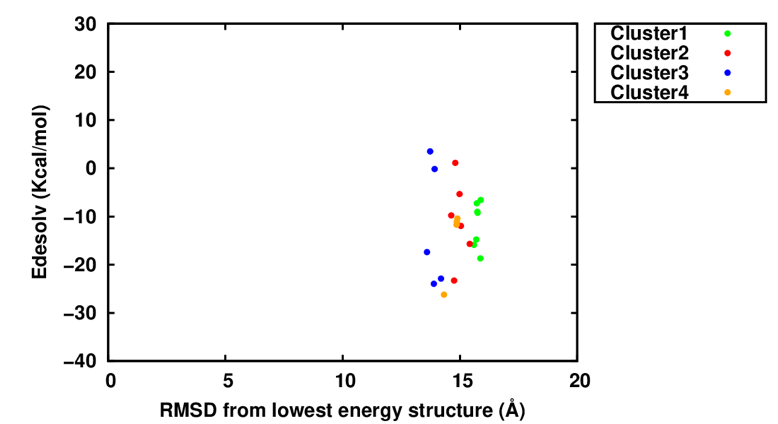


**Figure S10.** Clusters distribution of the Sod1 docking calculation with ECs only at P 0.50. **A**) HADDOCK score. **B**) Edesolv scoring function.

|  | **L-asparaginase II “ECs + NMR”** | | | | | |
| --- | --- | --- | --- | --- | --- | --- |
|  | **D = 10 Å** | | | **D = 12 Å** | | |
| **Prob** | **TP+FP** | **TP** | **PPV** | **TP+FP** | **TP** | **PPV** |
| **0.9** | 3 | 3 | 1.0 | 3 | 3 | 1.0 |
| **0.85** | 3 | 3 | 1.0 | 3 | 3 | 1.0 |
| **0.8** | 3 | 3 | 1.0 | 3 | 3 | 1.0 |
| **0.75** | 4 | 4 | 1.0 | 3 | 3 | 1.0 |
| **0.7** | 4 | 4 | 1.0 | 4 | 4 | 1.0 |
| **0.65** | 4 | 4 | 1.0 | 4 | 4 | 1.0 |
| **0.6** | 6 | 5 | 0.8 | 4 | 4 | 1.0 |
| **0.55** | 8 | 7 | 0.9 | 4 | 4 | 1.0 |
| **0.5** | 8 | 7 | 0.9 | 4 | 4 | 1.0 |
| **0.45** | 8 | 7 | 0.9 | 4 | 4 | 1.0 |
| **0.4** | 12 | 10 | 0.8 | 5 | 5 | 1.0 |
| **0.35** | 14 | 12 | 0.9 | 7 | 7 | 1.0 |
| **0.3** | 17 | 14 | 0.8 | 9 | 8 | 0.9 |
| **0.25** | 28 | 21 | 0.7 | 19 | 15 | 0.8 |
| **0.2** | 43 | 31 | 0.7 | 34 | 26 | 0.8 |

**Table S1.** Interface residues identified using cutoff D = 10 Å and D = 12 Å for the L-asparaginase II protein.
